# Deep neural networks trained for estimating reflectance and illumination achieve lightness constancy differently than human observers

**DOI:** 10.1167/jov.26.2.11

**Published:** 2026-02-18

**Authors:** Alban Flachot, Jaykishan Patel, Thomas S. A. Wallis, Marcus A. Brubaker, David H. Brainard, Richard F. Murray

**Affiliations:** 1 Department of Psychology and Centre for Vision Research, York University, Toronto, Ontario, Canada; 2 Centre for Cognitive Science and Institute for Psychology, Technical University of Darmstadt, Germany; 3 Center for Mind, Brain and Behavior (CMBB), Universities of Marburg, Giessen, and Darmstad, Darmstadt, Germany; 4 Department of Psychology, University of Pennsylvania, Philadelphia, Pennsylvania, USA; 5 Department of Electrical Engineering and Computer Science, York University, Toronto, Ontario, Canada

**Keywords:** lightness constancy, intrinsic image decomposition, deep learning, 3D rendering, computational modeling

## Abstract

Lightness constancy, the ability to create perceptual representations that are strongly correlated with surface reflectance despite variations in lighting and context, is a challenging computational problem. Indeed, it has proven difficult to develop image-computable models of how human vision achieves a substantial degree of lightness constancy in complex scenes. Recently, convolutional neural networks have been developed that are proficient at estimating reflectance, but little is known about how they achieve this, or whether they are good models of human vision. We examined this question by training a convolutional neural network to estimate reflectance and illumination in a computer-rendered virtual world, and evaluating both the convolutional neural network and human observers in a lightness matching task. In several conditions, we eliminated cues potentially supporting lightness constancy: local contrast, shading, shadows, and all contextual cues. We found that the network achieved a high degree of lightness constancy, outperforming human observers. However, we also found that eliminating cues affected the convolutional neural network and humans very differently. Humans were most affected when local contrast cues were made uninformative, whereas the convolutional neural network mostly relied on shading and shadows. In a follow-up experiment, we found that the convolutional neural network could learn to exploit noise artifacts typically associated with ray tracing and correlated with illuminance, with potential implications for the many studies relying on ray-traced images. We conclude that convolutional neural networks can learn an effective, global strategy of estimating lightness, which is closer to an optimal strategy for the ensemble of scenes we studied than the computation used by human vision.

## Introduction

Lightness constancy is the ability to create perceptual representations that are strongly correlated with the achromatic surface reflectance^1^ of objects, across a wide range of lighting conditions and contexts. Like color constancy, this feature of our visual system helps us to build a stable and robust representation of our environment (Anderson, 2011), which is essential for high level tasks such as object recognition (Foster, 2011; Hurlbert, 2007; Murray, 2021). However, despite a long tradition of empirical and theoretical studies, it remains unclear what strategy the visual system uses to perform this feat, and for a simple reason: lightness perception is complex and depends on many different features, from low to high level, and from local to global. Some studies have shown the importance of local contrast in human lightness constancy (Adelson, 2000; Arend, Reeves, Schirillo, & Goldstein, 1991; Arend & Spehar, 1993; Foster, 2011; Gilchrist, 2006; Kraft & Brainard, 1999; Wallach, 1948; Wallach, 1963; Wedge-Roberts et al., 2020). Others have shown that global contextual features can greatly influence human lightness and color judgment, including scene complexity (Kraft, Maloney, & Brainard, 2002), object shading and shadows (Szafir, Sarikaya, & Gleicher, 2015), luminance and chromaticity range (Adelson, 1993; Webster & Mollon, 1995), and specular highlights (Wedge-Roberts et al., 2020). Additional studies have shown the relevance of high-level features such as memory and familiarity, particularly in the natural world (Granzier & Gegenfurtner, 2012; Tsuda, Fujimichi, Yokoyama, & Saiki, 2020). A consequence is that, despite many attempts (Dakin & Bex, 2003; Funt, Ciurea, & McCann, 2000; Land & McCann, 1971; Murray, 2021), an accurate and image-computable model of this phenomenon has yet to be successfully developed.

Recent studies have shown that deep neural networks (DNNs) are a promising approach to performing many visual tasks (Krizhevsky, Sutskever, & Hinton, 2012; Storrs, Anderson, & Fleming, 2021; Yao, Lin, Xia, Zhao, & Zhou, 2020). In particular, DNNs have made tremendous progress toward solving illumination estimation for complex and naturalistic images (Afifi, Brubaker, & Brown, 2022; Ulucan, Ulucan, & Ebner, 2024). Furthermore, deep learning models for intrinsic image decomposition have been successful at extracting the physical surface reflectance of objects in complex scenes (Fan, Yang, Hua, Chen, & Wipf, 2018; Heidari-Gorji & Gegenfurtner, 2023; Li, Shafiei, Ramamoorthi, Sunkavalli, & Chandraker, 2020; Li & Snavely, 2018b; Wang, Philion, Fidler, & Kautz, 2021; Yu & Smith, 2019). DNNs trained for color classification and color constancy have also been shown to develop supra-human levels of constancy and human-like representations of color (Flachot et al., 2022). And recently, Murray, Brainard, Flachot, and Patel (2023) showed how these models can be tested for lightness constancy on the same images as human observers, allowing for a direct and behaviorally relevant comparison. DNNs are, therefore, interesting candidates for producing a useful, image-computable model of human lightness constancy.

In this study, we aimed to extend our understanding of human lightness constancy via a series of experiments, while exploring the potential of a recent and promising family of deep learning algorithms as models of human lightness constancy. We address three main questions: 1) What image properties, such as local contrast or shadow-like edges, do human observers rely on for lightness constancy in images of complex scenes? 2) Can DNNs, and in particular those trained for intrinsic image decomposition, achieve human levels of lightness constancy on the same images? 3) And perhaps most important, do these networks rely on the same image properties in the same way as human observers?

To address these questions, we devised a lightness matching experiment and rendered images of three-dimensional scenes to test both human participants and a DNN trained for intrinsic image decomposition. In several different conditions, we manipulated cues in the rendered scenes that previous work has found to be relevant to lightness and color constancy for human observers (Gilchrist, 2006; Kraft & Brainard, 1999; Szafir et al., 2015). This allowed us to identify what image properties human observers and the DNN rely on for lightness constancy.

We found that the DNN was able to achieve a high degree of lightness constancy in complex scenes, and in fact better than human observers. Furthermore, there were some notable similarities between human and DNN performance, such as partial lightness constancy in which reflectance matching errors increased linearly as a function of illumination changes. This finding is broadly consistent with previous work suggesting that important phenomena in human lightness perception, such as lightness illusions and partial constancy, may not simply be ‘failures’ of perception, so much as byproducts of a strategy of relying on natural scene statistics to interpret deeply ambiguous retinal images (Allred & Brainard, 2013; Murray, 2020; Patel et al., 2025).

However, we also found some important differences between human observers and the DNN. The image properties that the DNN relied on differed substantially from those used by humans. Human observers relied largely on local contrast, whereas the DNN was able to exploit global properties such as cast shadows. In a follow-up experiment, we also showed that the DNN could achieve lightness constancy by exploiting a common rendering artifact associated with ray tracing, whereas this artifact did not affect human observers’ performance. These differences suggest some future directions for developing DNN models of lightness constancy, which we return to in the General discussion.

## Experiment with human observers

### Methods

#### Observers

Nine observers participated in the experiment. All were naive except one, who was the first author. Five were male, four were female, and ages ranged from 25 to 34 years. All reported normal or corrected-to-normal vision, and gave written informed consent. All procedures were approved by the Office of Research Ethics at York University.

#### Stimuli

**Figure 1.**
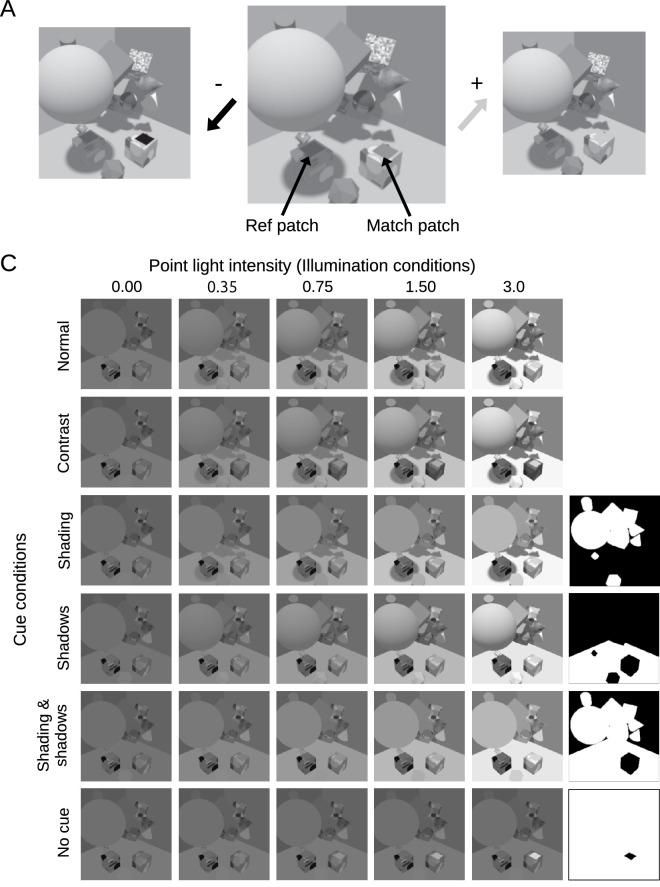
Stimulus images in the lightness matching experiment. (A) A typical trial: an image was shown with a reference patch and a match patch (center). The task of the observer was to adjust the reflectance of the match patch to match that of the reference patch. The match reflectances shown at left and right are extreme values, chosen for illustration purposes. (B) Point light intensities and scene cue manipulations. Each column shows stimuli in a single lighting condition, with the indicated value of point light intensity. Each row shows stimuli in a different cue condition, where we manipulated contrast, shadow, and shading cues. The black-and-white images to the right of the bottom four rows are masks that we used to create the stimuli in these conditions, as described in the main text.

We rendered 256 × 256 pixel images with three color channels (RGB) using the EEVEE engine in Blender 2.92 (Figure 1A) (Blender 2.92 Documentation Team, 2021). We rendered all surfaces using a Lambertian (i.e., matte) material model, so each surface was characterized by three reflectance values, one for each chromatic channel. When describing rendered stimuli, we use the term ‘RGB reflectance’ to mean the triplet of simulated reflectances assigned to a surface in the three color channels, in the scene description provided to the renderer. The rendered scenes had two walls and a floor, with 14 objects of different shapes, RGB reflectances, sizes, and positions, randomly sampled from the distribution we used for training the DNN (see Training images). Each image showed the same arrangement of geometric objects. The RGB reflectance assigned to each object was the same in all images, except for a small number of test objects, where it varied from image to image as described below. To speed rendering and introduce some variability into the stimuli, we rendered RGB images with different randomly chosen reflectances in the three color channels, and used each channel of the rendered image as a separate achromatic stimulus in the experiment. As a result, there were three subsets of stimulus images that showed objects with different achromatic reflectances. We refer to each single-channel image as a ‘luminance’ image, because it was used to generate the on-screen luminance. (Thus, the luminance image was not a weighted sum of the color channels, as is sometimes the case.) For each RGB image, Blender also rendered an image that gave the RGB reflectance at each pixel. *Illuminance* is incident luminous flux per unit area (e.g., lumens/m^2^ in SI units), and for a Lambertian surface it is proportional to the ratio of luminance and reflectance (McCluney, 2014). For each stimulus, we calculated an ‘illuminance image’ that indicated the amount of incident light at each location, by taking the ratio of the luminance and achromatic reflectance images at each pixel.

We chose to exclusively use Lambertian surfaces to avoid specular highlights and limit the linear dynamic range of our images to the standard 8-bit range without requiring non-linear tone mapping. This choice has the benefit of avoiding biases in the encoding of luminance in our images, but comes with a lack of realism. We describe some potential limitations of this approach in the Discussion.

Every stimulus included a *reference cube* and a *match cube*. On top of the reference cube was the *reference patch*, and on top of the match cube was the *match patch*. The observer’s task was to adjust the reflectance of the match patch to match that of the reference patch, as illustrated in Figure 1.

Each scene had two achromatic simulated light sources: an ambient light, with intensity fixed at 0.35 in Blender’s intensity units (IU), and a point light located above, with intensity set to 0.00, 0.35, 0.75, 1.50, or 3.00 IU, as shown in Figure 1B. The point light was located outside the visible part of the scene, so viewers had only indirect information about that light source, from shading and shadows. The reference patch reflectance was 0.20, 0.40, or 0.60. The match patch reflectance ranged from 0.000 to 1.000, in increments of 0.025. We rendered stimuli with all combinations of these scene parameters: 5 point light intensities, 3 reference patch reflectances, and 41 match patch reflectances.

The reference cube and patch were located under a large sphere (Figure 1) and thus were not affected by the point light source, unlike the match patch. When the point light intensity was greater than zero, the reference and match patches were thus under two different illuminations. This configuration allowed us to study lightness constancy using one stimulus per trial, placing our study in the special case of lightness constancy in complex scenes, where the illumination changes within the image (Murray, 2021). This makes sure that simple adaptation mechanisms are not enough to solve our task—as we confirm later in the modeling section.

To vary the availability of cues that could potentially support lightness constancy, we created six variations of these stimuli (Figure 1B). 1) In the normal condition, the stimuli were rendered as described. 2) In the contrast condition, the reflectance of the match cube was chosen such that the average luminance of the top face of the match cube (surrounding the match patch) was the same in every image, and equal to the average luminance of the top face of the reference cube (surrounding the reference patch). As a result, matches based on local contrast would equate the luminance of the reference and match patches, not their reflectances. This manipulation silenced local contrast as a cue to reflectance.

In the remaining four conditions, the stimuli were created by taking images from the normal condition, and replacing a subset of pixels by corresponding pixels from normal images where only ambient light illuminated the scene (Figure 1B, top left, labelled normal and 0.00). The white pixels in the black-and-white images on the right in Figure 1B indicate which pixels were replaced in each condition. The values of the inserted pixels were scaled so that their mean was the same as that of the pixels they replaced. 3) In the shading condition, pixels depicting the 14 random geometric objects were replaced by pixels from ambient-illuminated images. This eliminated shading and shadows on the 14 objects. Cast shadows on the walls and floor were preserved. 4) In the shadows condition, all pixels depicting the floor were replaced by corresponding pixels from ambient-illuminated images. This eliminated cast shadows on the floor. 5) In the shading & shadows condition, all pixels depicting the 14 geometric objects and the floor were replaced by pixels from ambient-illuminated images. Only pixels depicting the reference and match cubes and the walls were left unchanged. Finally, 6) in the no-cue condition, all pixels except those depicting the reference and match patches were replaced by corresponding pixels from ambient-illuminated images. This was a limiting condition, where there were no cues to the simulated lighting, and in this condition we expected observers to simply match luminance instead of reflectance, that is, to show no lightness constancy. The average luminance around the reference and match patches was the same in the contrast and no-cue conditions.

In summary, the experiment had three reference conditions (one for each reference reflectance value), five illumination conditions (one for each point light intensity value), and six scene cue conditions (from normal to no-cue).

The stimuli were back-projected onto a 100 cm wide × 70 cm high screen with a ProPixx 1,440 Hz projector in a dark room. Observers sat 120 cm from the screen, and head position was stabilized with a chinrest. The projected stimulus image was 25 cm square, and subtended 11.9 degrees of visual angle, horizontally and vertically. The image was surrounded by a gray background projected onto the screen. We made luminance characterization measurements from the display using an Xrite i1Display Pro colorimeter, fitted a gamma-correction model, and transformed the stimuli using custom software so that displayed luminance was proportional to rendered luminance (Brainard, Pelli, & Robson, 2002). We scaled the rendered stimuli before display so that luminance ranged up to 125.4 cd/m^2^. We created the experiment using Python 3.8 and PsychoPy 2024.1.4 (Pierce et al., 2019). Python code for generating stimuli, running the experiment, and analyzing the results can be found in our GitHub repository.^2^ Links to download the full set of stimuli are also provided.

#### Procedure

On each trial, the observer viewed an achromatic stimulus randomly chosen from one of the six cue conditions (normal, contrast, shading, shadows, shading & shadows, and no-cue). Images from the three rendered color channels were randomly interleaved. The simulated point light intensity was randomly set to 0.00, 0.35, 0.75, 1.50, or 3.00 IU. The reference patch reflectance was randomly set to 0.20, 0.40, or 0.60. The match patch reflectance was randomly set to an initial value between 0.00 and 1.00. The observer was instructed to match the appearance of the match patch to that of the reference patch. The observer used a mouse scroll wheel to adjust the reflectance of the match patch, in steps of 0.025 on the scale from 0 to 1 that we used to specify reflectance, until they judged it to have the same lightness as the reference patch. For each reflectance of the match patch, the software loaded and displayed a new rendered image. There was no limit on response time, although the observer was told they would be evaluated based on both accuracy and speed, so they were incentivized to make quick, visual match settings instead of cognitively inferred settings. When the observer was satisfied with the match, they clicked the mouse, and after a brief pause (0.3 second) the next trial began.

There were six conditions, five point light intensities, three reference reflectances, and three color channel to grayscale conversions, for a total of 6 × 5 × 3 × 3 = 270 trials. These trials were interleaved with additional trials showing similar stimuli rendered with the Cycles engine in Blender 2.92, for a comparison we describe below; see The risks of ray-traced rendering for training models of lightness constancy. Despite the inter-trial interval of 0.3 second, it occasionally happened that the mouse button press that ended one trial had not been released when the next trial began, in which case the initial, randomly chosen match reflectance was saved as a match, with a response time near zero. These trials amounted to 1.5% of the total, and were not included in the analyses. On average, valid trials had a response time of 7.1 seconds, and the whole experiment took 42 minutes.

#### Analysis

We quantified lightness constancy using the standard constancy index called the Thouless ratio (Thouless, 1931), defined as follows. Let *r*_1_ be the reflectance of the reference patch in a matching task, and let *r*_*m*_ be the reflectance that the observer chooses at the match patch. Let *i*_1_ and *i*_*m*_ be the illuminances at the reference and match patches, respectively. An observer who simply matches luminance (i.e., has no lightness constancy) chooses the match reflectance *r*_0_ where *r*_0_*i* = *r*_1_*i*_1_, which implies *r*_0_ = *r*_1_*i*_1_/*i*. The Thouless ratio τ is defined as

(1) $$\begin{array}{c} \tau =\frac{\log r_{m}-\log r_{0}}{\log r_{1}-\log r_{0}}.\; \end{array}$$

A perfectly lightness-constant observer (*r*_*m*_ = *r*_1_) has a Thouless ratio of τ = 1, and a luminance-matching observer (*r*_*m*_ = *r*_0_) has τ = 0. The logarithms in this definition reflect the fact that perceived lightness is a monotonic, compressive function of physical reflectance. The Thouless ratio is similar to the color constancy index that is often used to quantify constancy in a multidimensional color space (Arend et al., 1991; Brainard, 1998).

Equation (1) can be rewritten as

(2) $$\log r_{m}=\left(\tau -1\right)\left(\log i_{m}-\log i_{1}\right)+\log r_{1}.$$

This shows that, for an observer with a fixed Thouless ratio τ, log match reflectance (log *r*_*m*_) is an affine function of log match illuminance (log *i*_*m*_), with slope τ − 1, and thus such a plot reveals their Thouless ratio τ. When the observer’s reflectance match is invariant across different illumination intensities (perfect constancy, τ = 1) the slope is 0. When the observer matches luminance (no constancy, τ = 0), the slope is −1. We calculated each observer’s Thouless ratio in each condition from the slope of a least-squares linear regression line fitted to the logarithm of the match reflectance settings as a function of the logarithm of illuminance at the match patch.

### Results

**Figure 2.**
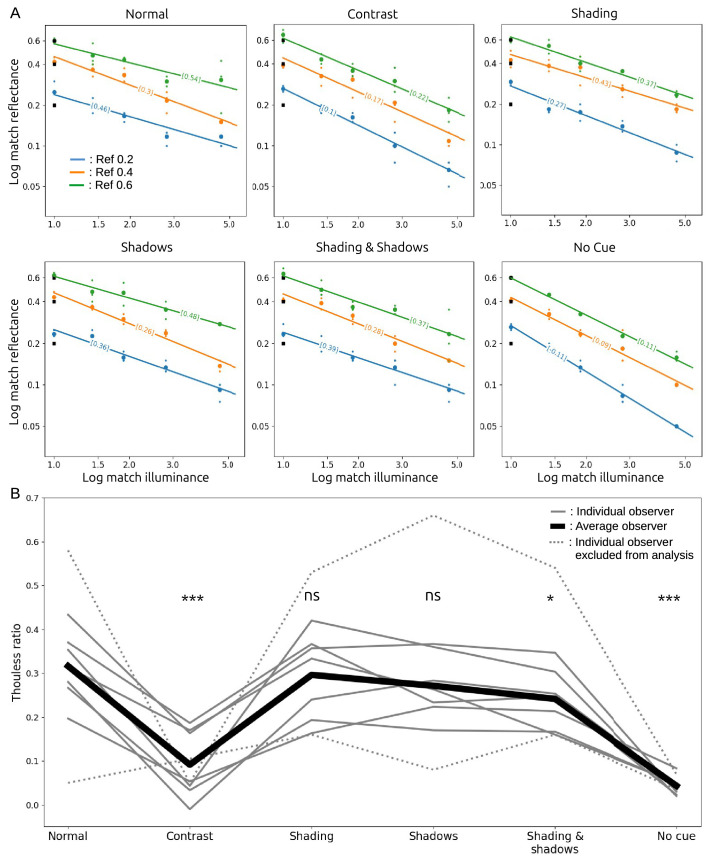
(A) Results for a typical observer in all conditions. Each small dot shows the result for a single trial, for a stimulus generated in one of the three RGB color channels, and each larger dot shows the average match setting across those three trials. Log match reflectance declined as an approximately linear function of log match illuminance. Bracketed values show Thouless ratios estimated from the fitted lines. (B) Thouless ratios for individual observers (grey lines) and the mean across observers (black line). Significance levels are the results of paired *t*-test comparisons to the Normal condition. *p < 0.05, **p < 0.01, ***p < 0.001.

Figure 2A shows raw data for a typical observer in all conditions. Each panel plots log match reflectance against log match illuminance (represented as a multiple of the illuminance at the reference patch), to test the linear relationship predicted by Equation (2). Each color shows results for a single reference reflectance. Each small dot shows the result for a single trial, for a stimulus generated in one of the three RGB color channels, and each larger dot shows the average match setting across those three trials. This panel confirms the affine relationship between log match reflectance and log match illuminance described by Equation (2): across all observers, conditions, and reference reflectances, the matches were well fit by linear regression, with an average coefficient of determination *R*^2^ = 0.92. The bracketed values on each line are the Thouless ratios estimated from the line’s slope as in Equation (2).

Constancy was approximately the same for the three reference reflectances. A one-way repeated measures analysis of variance (ANOVA) testing for an effect of reference reflectance on Thouless ratio did not reach significance, *F*(2, 12) = 0.53, *p* = 0.69. However, matches were slightly different for images from the three color channels. A one-way repeated-measures ANOVA of the effect color channel on match reflectance was significant, *F*(2, 12) = 69.8, *p* < 10^−6^. Nevertheless, the differences were small: the average matches for the R, G, and B channel stimuli were 0.25, 0.26, and 0.28, respectively. Small differences in lightness matches between these groups of stimuli are not surprising, as they had different reflectances assigned to walls and objects.

Figure 2B shows Thouless ratios for all observers in all conditions. Light gray lines show results for individual observers, averaged over reference reflectances, and the heavy black line shows the average over observers and reference reflectances. The two dotted lines show results for two observers we excluded from the analysis. The lower dotted line indicates poor constancy in all conditions, and so the observer may not have understood the task. The upper dotted line was from the first author, a highly practized and non-naive observer who had unusually good constancy in some conditions (but nevertheless a qualitatively similar profile across conditions, compared with the other observers).

Overall, Thouless ratios were relatively low, with an average of 0.32 in the normal condition. It is not unusual to find weak constancy when stimuli are not rich, naturalistic scenes (Katz, 1935). Here the stimuli were artificial Lambertian scenes, with simple lighting conditions, shown on a two-dimensional display, and these factors may have contributed to relatively weak constancy.

The pattern of Thouless ratios across conditions was quite consistent across observers: highest constancy in the normal condition; little change in the shading, shadows, and shading & shadows conditions, suggesting that observers were largely indifferent to object shading and shadows (after the experiment, several observers reported being unaware that shadows were sometimes absent); lowest constancy was found in the no-cue condition, as expected, with an average Thouless ratio of 0.04; and low constancy in the contrast condition, with an average Thouless ratio of 0.09. These observations strongly suggest that, for our stimuli, the perceived color of the match patch is mostly influenced by local contrast. The asterisks in Figure 2B show the results of paired *t*-tests that compared each condition with the normal condition, which support these qualitative observations.

Although there were individual differences, most observers exhibited a similar behavioral pattern, namely low sensitivity to shading and shadows, and high sensitivity to local contrast. In the following section, we test whether the same results can be found with a DNN trained to estimate the reflectance of objects independently of lighting conditions and context.

## Deep learning models

We trained a DNN for intrinsic image decomposition, and evaluated it for lightness constancy by simulating its performance on the same task we studied with human observers.

### Training images

**Figure 3.**
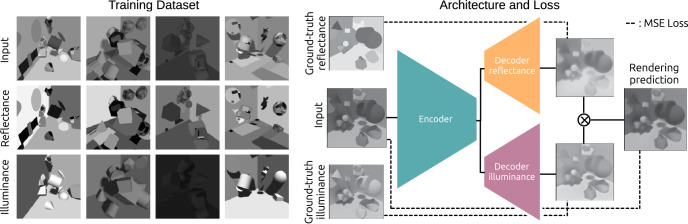
Training procedure for the convolutional neural network. (*Left*) Examples of training images. (*Right*) Architecture and loss.

We rendered 121K training images and 7K validation images using the EEVEE engine in Blender 2.92 (Blender 2.92 Documentation Team, 2021). Each scene included 17 randomly chosen geometric shapes (spheres, rectangular prisms, cylinders, tori, and icosahedra), with randomized locations, orientations, and sizes (Figure 3). Each object was rendered with a surface pattern that was randomly chosen to be a) a solid color, with a reflectance uniformly sampled between 0.05 and 0.90, which is approximately the range of reflectances found in natural scenes (Gilchrist, 2006), b) a Voronoi pattern, c) a low-pass noise pattern, or c) a random composite of filled-in rectangles and ellipses, again with reflectance uniformly sampled between 0.05 and 0.90. The scenes were illuminated by an ambient light source with a random intensity and a directional light source with random direction and intensity. Both light sources had randomized colors. The virtual camera was placed at a randomly chosen location and was directed at the center of the scene. We also rendered ground-truth reflectance images of each scene, and calculated illuminance images as the pixelwise ratio of luminance and reflectance images. Images were saved in EXR format with 32-bit floating point precision. The GitHub repository shared previously includes the Python code that generated these images, as well as a link to download the full training set.

We rendered the images in color, but used the three color channels as separate achromatic training images. We also augmented the training data using vertical and horizontal flips. These augmentations produced a training set with 121K × 3 channels × 4 flips = 1.45 M achromatic images for each of luminance, reflectance, and illuminance.

### Model, loss, and training

The network had a modified U-Net architecture with one encoder, and two separate decoders for reflectance and illuminance (Figure 3B) (Li et al., 2020). The encoder had 2,240 convolutional filters split over 6 convolutional layers. Both decoders had 1,408 convolutional filters, also split over six convolutional layers. Each layer of the encoder sent its output to the next encoder layer in a feedforward manner, and also to the decoders via direct skip connections.

We trained the model to map training images to their corresponding ground-truth reflectance and illuminance images. The cost function for each estimate was

(3) $$L_{C}=\frac{1}{N}\sum_{k=1}^{N}{\left(P_{C,k}-G_{C,k}\right)}^{2},$$

where *P* is the model’s prediction, *G* is the ground-truth image, *C* stands for either *reflectance* or *illuminance*, and *N* is the number of pixels in the image.

Because the scenes depicted Lambertian surfaces, each input image *I* was the product of its two intrinsic components, reflectance and illuminance. As a regulatory term, we imposed a reconstruction cost by penalizing any discrepancy between the input image and the pointwise product of the model’s reflectance and illuminance predictions. We defined the reconstruction loss as

(4) $$L_{rec}=\frac{1}{N}\sum_{k=1}^{N}{\left({\tilde{I}}_{k}-I_{k}\right)}^{2}.$$

where $\tilde{I}$ is the pixelwise product of the model’s estimates of reflectance and illuminance, *I* is the input image, and the sum is over image pixels.

The total loss $L_{R}$ was a combination of three terms:

(5) $$L_{R}=\alpha L_{reflectance}+\beta L_{illuminance}+\gamma L_{rec},$$

where α, β, and γ are scalars. Because we evalauted models on their ability to estimate reflectance, we favored learning reflectance estimation over the other criteria by setting α = 4 and β = γ = 1. The illuminance and reconstruction losses served as regulatory cost functions.

Training lasted for 60 epochs, and in each epoch the network was trained on 125K images × 3 channels = 375K images. Each image was flipped horizontally and vertically with a probability of 0.5 for each direction. We used the Adam optimizer with a learning rate that started at 5 × 10^−5^ and decayed by a factor of 2 every 10 epochs. The loss flattened out around the 50th epoch. The GitHub repository shared elsewhere in this article provides code that implements the network and training routines.

Because the training procedure included several random steps, such as the initialization of the weights and the order in which images were shown, different training runs resulted in different learned weights. We performed three training runs, resulting in three different training instances.

### Validation

**Figure 4.**
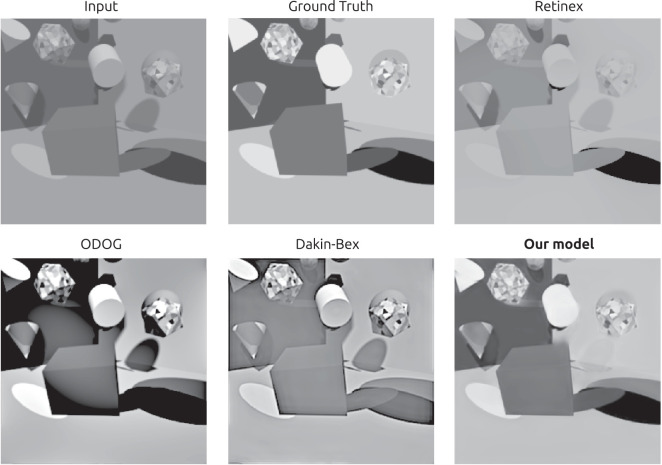
Example of an image from the validation set, its ground-truth reflectance, and the predictions from the models tested. The DNN's prediction were the closest to the ground-truth, and it was the only model able to largely eliminate shading and shadows.

**Figure 5.**
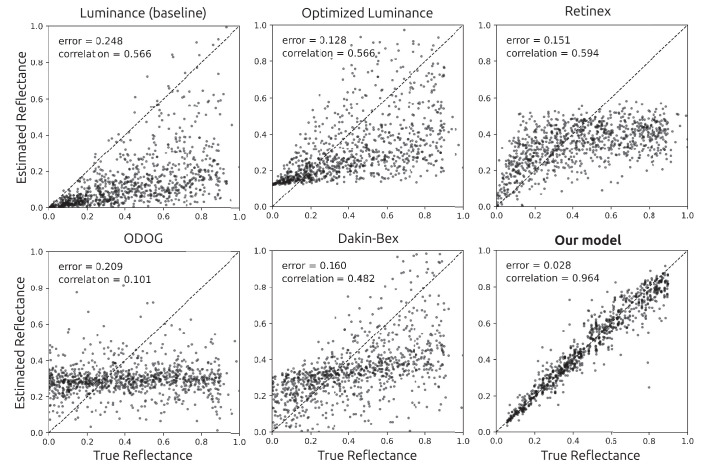
Scatter plots of models’ reflectance estimates against the ground-truth reflectance. The DNN model had the best performance of the models tested. The reported errors are the median absolute prediction error across pixels.

After training, we evaluated the network’s ability to estimate reflectance on the validation set, which consisted of images that were generated by the same random procedure that created the training images, but that were not used during training. For these validation tests, the network was only provided with the luminance images and not the ground truth for reflectance or illuminance. We found that the reflectance output of the network largely removed shading and shadows from input images (Figure 4). Furthermore, the network produced generally good pixelwise reflectance estimates, with a median absolute error of 0.028, measured in reflectance units that range from 0 to 1 (Figure 5).

For comparison, we also evaluated a number of alternative models. There are few image-computable lightness models in the psychophysics literature, so we evaluated models of both lightness (perceived reflectance) and brightness (perceived luminance). We evaluated ODOG, which is a brightness model (Blakeslee & McCourt, 1999), retinex, which is a lightness model (Funt et al., 2000; Land & McCann, 1971), and the Dakin-Bex normalization model, which has been described in both ways (Dakin & Bex, 2003). These models do not give outputs in reflectance units, so we optimized their performance by finding the affine transformation that minimized each model’s median absolute error when predicting reflectance in the training set, as follows. We applied each model to the 128K images in the training set. We found the affine transformation that gave each model the lowest median absolute error when predicting reflectance at 10^3^ pixels randomly sampled from each training image, for a total of 1.28 × 10^8^ pixels. We then used these optimal affine transformations, estimated from the training set, to map model outputs to predicted reflectances on the validation set. As baselines, we also tested two null models whose outputs were in one case simply the luminance input, and in the other case the luminance output modified by an optimal affine transform as with the other models.

Unlike the DNN, the outputs of these alternative models generally did not remove shading and shadows (Figure 4). Furthermore, even after an optimal affine transform of their outputs, they had substantially higher prediction error than the DNN (Figure 5). In fact, the three lightness/brightness models (ODOG, Dakin-Bex, and retinex) gave reflectance estimates whose median absolute errors were even higher than that of the optimized luminance model, whose output was simply an affine transformation of the luminance input. Patel et al. (2025) found similar results for a trained network and classical models, using a similar but not identical network and training set. The poor performance of classical models on our stimuli may not be surprising, in that they were largely developed for and tested on relatively simple two-dimensional stimuli, where they are often successful at predicting human lightness and brightness judgments (Nedimović, Zdravković, & Domijan, 2022).

The validation results show that training was successful, in that the network was able to decompose rich three-dimensional scenes into their reflectance and illuminance intrinsic image components, at least when the scene was typical of the training distribution. We also found that the network performed better than classical models of lightness and brightness. As such, the network is an interesting candidate for a model of human lightness constancy. In the following section, we evaluate the network in the same matching task we used with human observers, for a more direct comparison.

### Lightness constancy in a matching task

To further evaluate the network’s degree of lightness constancy and compare it with that of human observers, we ran it in a simulation of the matching task performed in the behavioral experiment reported elsewhere in this article. In each cue condition of the simulated experiment, and for each point light intensity and reference reflectance, we found the network’s match setting as follows. We applied the network to the same stimuli viewed by human observers, with a fixed reference patch reflectance and with a range of match patch reflectances. We took the network’s match setting to be the match patch reflectance for which the network’s mean output was the same at the reference patch and the match patch. For the finite set of test stimuli, the model’s output was never exactly the same at the reference and match patches, so we found the match setting by interpolation or extrapolation. We then calculated a Thouless ratio for the network, just as we did for human observers, from a fit of Equation (2) to the logarithm of the network’s match reflectance setting as a function of log illuminance at the match patch.

As in the previous section, the DNN had only the luminance image as an input, and was not provided with ground-truth reflectance or illuminance images at inference time. This point, coupled with the fact that human observers had access to the entire stimulus image well within their visual field, with no required fixation point or fixed display time, allowed for as fair a comparison as possible between our networks and our human observers. We note, however, that our supervised training regime means that the network learned from pixelwise ground truth for reflectance and illuminance at training time, which is unlike how human observers develop visual abilities (see Discussion).

**Figure 6.**
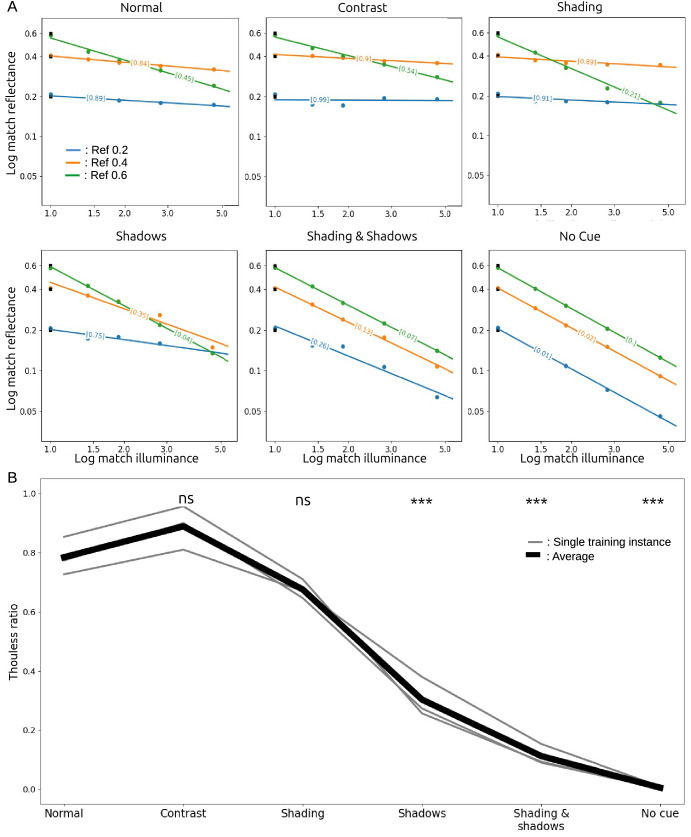
(A) Results for one training instance of the network in all conditions. (B) Thouless ratios for single training runs (gray lines, *N* = 3) and the average across training runs (black line). Results from different training runs were similar, and tend to overlap in the figure. Significance levels are the results of paired *t*-test comparisons to the Normal condition.

In all stimulus conditions, the networks’ log match reflectance was an approximately linear function of log illuminance (Figure 6A). This finding is itself noteworthy, and an important similarity to human observers that did not have to be the case for such a complex and highly nonlinear network. This linearity also means that the Thouless ratio is a meaningful metric for describing the network’s performance in the matching task. The Thouless ratio for each condition is shown in brackets on each fitted line.

Figure 6A also shows that this training instance’s match settings were largely constant as a function of illuminance in the normal and contrast conditions, and that they varied with illuminance in the remaining conditions. This result differs from what we found for human observers. Additionally, the Thouless ratios varied markedly with the reference reflectance (a one-way repeated measures ANOVA revealed an effect of reflectance on match reflectance) *F*(2, 12) = 102.3, *p* = 0.0003, with much weaker constancy when the reference reflectance was 0.6. A partial explanation of this difference can be found in the network’s illumination predictions (not shown here). When the reference reflectance was 0.6, the model assigned an unusually high illuminance to the reference cube. What led the network to make this mistake, however, is unclear.

Figure 6B shows the mean Thouless ratio for each condition, averaged across reference reflectances for each training instance (gray lines) and averaged across training instances and reference reflectances (bold black line). As was the case for human observers, the network’s constancy was affected by the contextual cue manipulations: a one-way repeated measures ANOVA revealed a significant effect of cue condition on Thouless ratio, *F*(5, 10) = 138.7, *p* ≪ 0.01. However, the similarity ends there, as some differences clearly emerge. First, in the normal condition, the Thouless ratio was much higher for the network (mean 0.79) than for human observers (0.38), indicating that the network had markedly better lightness constancy than humans in the realistic baseline condition. (Note that the *y*-axis ranges are different in Figures 2B and 6B.) Second, the network’s performance did not suffer in the contrast condition, in which the local contrast cue was silenced (a *t*-test comparison of Thouless ratios in the normal and contrast conditions gave *p* = 0.23), whereas human observers’ lightness constancy was dramatically worse in the contrast condition. Third, the network’s performance was substantially worse in the shadows and shading & shadows conditions than in the normal condition, whereas human observers performed similarly and only mildly worse, respectively, in those conditions. Fourth, differences across training instances were smaller than individual differences between human observers.

**Figure 7.**
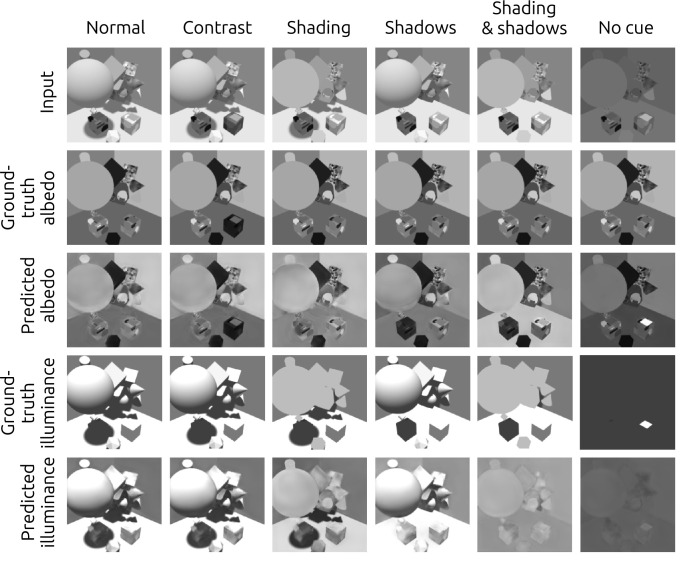
Examples of the network’s output in all six conditions. Except for the match cube in the no-cue condition, the ground-truth reflectance is the same in all conditions, unlike illuminance. The model misjudged reflectance in the physically inconsistent conditions. This is particularly obvious in the no-cue condition, where the model attributed the high luminance of the match patch to a high reflectance instead of a high illuminance.

Visual inspection of the model’s output supports some of these findings and gives insight into the model’s performance. Figure 7 shows typical stimuli in all conditions, in the case where the point light had high intensity, resulting in strong cast shadows. The figure also shows ground-truth and network predictions for reflectance and illuminance. There are clear differences in the model’s output across conditions. In the normal and contrast conditions, the model correctly inferred lower illuminance at the reference cube (which was in shadow) than at the match cube, and correctly assigned approximately the same reflectance to the reference and match patches. In the shadows and shading & shadows conditions, however, the network assigned approximately equal illuminance to the reference and match cubes, and assigned a much higher reflectance to the match patch than to the reference patch. Finally, in the no-cue condition, as expected, the model inferred almost uniform illuminance everywhere, and again attributed a much higher reflectance to the match patch than to the reference patch.

These results suggest that the network had some ability to model relationships over large image regions, for example, using cast shadows to infer reflectance at the reference and match patches. The network seemed not to rely on local contrast to estimate reflectance, but instead was able to estimate local lighting conditions, taking into account features such as shading and cast shadows. As a result, the network was susceptible to some stimulus manipulations in ways that human observers largely were not. For example, when we eliminated the shadows on the floor in the shadows condition, the network’s estimate of local lighting conditions changed, and so did its estimates of reflectance.

Overall, these results show that the network learned to use cues such as shading and shadows to disentangle the reflectance and illuminance components of its input. By doing so, it achieved a much higher level of lightness constancy than human observers on the same images. It also managed to learn the non-trivial relationship between distant objects (such as the large sphere) and the shadows they cast on other objects (such as the floor and reference cube). This last feature, however, is fragile: small changes in the image can disrupt it, as when we removed the sphere’s shading in the shading condition or the sphere’s shadow in the shadows condition.

Most important, the image properties that the network relied on differed from those used by human observers: although the network mostly relied on shadows, human observers’ judgements in these scenes were mostly modulated by local contrast. Thus, although the network can use naturalistic cues, its strategy for inferring reflectance is quite unlike that of human observers on these images. This finding is consistent with the findings reported in the next section, where we demonstrate some pitfalls to be mindful of when using deep learning models of human behavior.

An important question for future work is whether these findings depend on the U-Net architecture we used. Preliminary work shows that a wide range of network architectures can generate similar lightness illusions (Patel, Flachot, Vazquez-Corral, Derpanis, & Murray, 2024). Whether they have similar lightness matching behavior, and rely on similar image properties to achieve lightness constancy, remains to be seen.

## The risks of ray-traced rendering for training models of lightness constancy

In the previous section, we saw that it is possible to train a convolutional network to disentangle the contributions of reflectance and illuminance to a luminance image, relying on naturalistic features such as shading and shadows. These networks effectively learned a strategy for lightness constancy based on physically relevant cues, and one that differs from the strategy used by human observers. This was only possible due to 1) the care we took in training the models using images that, to the best of our knowledge, do not contain information other than the stipulated cues that the models could use to solve the task and 2) using test images that were novel, in the sense that these exact images were not seen by the model in training, but fell within the training distribution (i.e., they were novel, independent, and identically distributed samples).

Our stimuli were rendered using rasterization instead of the more popular ray-tracing approach. And for good reason: datasets rendered with ray tracing can present artifacts, and it is well-known that deep learning models, and in particular those trained via supervised learning, are prone to learn undesirable shortcuts, for example, based on biases or artifacts in the training set (Geirhos et al., 2020). In the following section, we document what artifacts can be present in datasets rendered with ray tracing, and how these are correlated with illuminance, with undesirable consequences for models trained for lightness constancy and intrinsic image decomposition in general.

### Ray-tracing noise as a confounding factor

**Figure 8.**
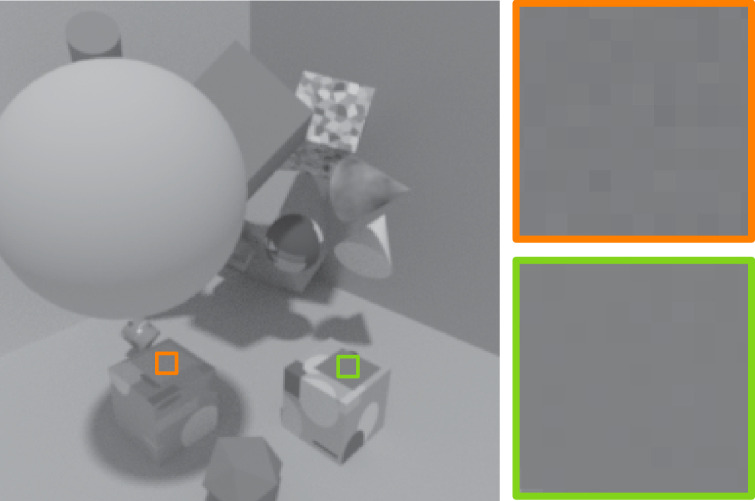
Example of image noise typically associated with ray tracing that can be a confounding factor for intrinsic image decomposition. The large squares on the right are zoomed-in views of the smaller squares in the rendered scene on the left. The two extracted patches in the image have roughly the same luminance, yet the patch in shadow (orange border) is noisier than the patch in direct light (green border). Even without a broader contextual context, this is enough local information to conclude that the patch on the left is less strongly illuminated, and thus shows an object with a higher reflectance.

Ray-tracing methods are more realistic than rasterization methods because they simulate physical light transport (Shirley & Morley, 2008). They tend to be the default rendering methods used to generate photorealistic images and in particular images used to train computer vision models. In fact, a large number of the large-scale synthetic datasets available for training models for intrinsic image decomposition were rendered using ray tracing methods (Li et al., 2021; Li et al., 2018; Roberts et al., 2021; Zhu et al., 2022). These methods’ advantage over rasterization is that they include a model of virtual light rays, *traced* back from the virtual camera to the virtual light source. However, their probabilistic nature is such that image regions with a more indirect path to the light source (e.g., regions in shadow) are less likely to be traced back, and thus tend to appear noisier. This is the case with Cycles, Blender’s ray-tracing algorithm (Blender 2.92 Documentation Team, 2021), but also many other standard ray-tracing algorithms. Although there are ways to reduce the amount of noise in an image, for example by increasing the sample count (the number of rays sampled in each pixel), areas in shadow will often nevertheless appear noisier. This correlation between illuminance and image noise is a potential confounding factor for lightness constancy, and is illustrated in Figure 8. Thus, we tested for an effect of this rendering noise in a lightness matching task, both for human observers and for the network that we examined above.

We rendered the same stimuli described in Methods, except that we used the Cycles renderer of Blender 2.92 instead of EEVEE. We used a sample count of 128, small enough that the rendering noise remains visible to the naked eye in shadowed areas. We call this new image set the *Cycles image set*.

### Experiment with human observers

We tested the same human participants as in the previous section, but here with the Cycles image set. As mentioned in Methods, the Cycles images were interleaved with a larger number of images rendered with EEVEE, for which results are reported above. In the Cycles conditions, we tested participants only in the normal and no-cue conditions. However, each condition included the same number of reference reflectances (*n* = 3), light intensities (*n* = 5), and color channels converted to achromatic luminance images (*n* = 3) as in the EEVEE image set. This provides a control for the main experiment: if human observers can exploit the rendering noise to perform the matching task, then there should be a significant improvement in the measured Thouless ratios in the two Cycles conditions.

**Figure 9.**
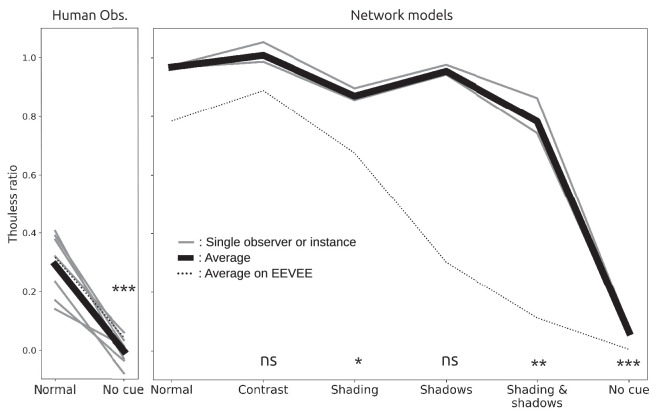
Thouless ratios, measured with the Cycles image set, for the seven human observers (left) and three training instances of the Cycles-trained model (right). Significance levels are the results of paired *t*-test comparisons to the normal condition. The faint dashed lines are the average previously found for the EEVEE dataset, shown here for comparison purposes.

That is not what we observed. There was a significant difference between the Thouless ratios measured for EEVEE and Cycles images (as shown by a repeated-measures ANOVA), *F*(1, 166) = 6.1, *p* = 0.014, but the effect was small and in the opposite of the hypothesized direction. Average Thouless ratios were 0.29 and 0.00 in the normal and no-cue conditions, respectively, for Cycles images, compared with 0.32 and 0.04 for the EEVEE images (Figure 9). This suggests that, if anything, the rendering noise in Cycles made the task slightly more difficult for human observers. Still, the differences are in any case very small in overall magnitude.

### Deep learning models

We also tested whether the DNN architecture we used for intrinsic image decomposition can exploit rendering noise as a cue to estimate reflectance. We rendered 128K new training images and 7K new validation images with the same rendering parameters as described in Training images, now using Cycles instead of EEVEE. For consistency, we used a sample count of 128. We also made three training runs of the network described above on this new image set, resulting in three training instances. We refer to the models trained on the EEVEE images as the *EEVEE models*, and the models trained on the Cycles images as the *Cycles models*.

We tested the Cycles models on the matching task in all conditions. We found a highly significant effect of the choice of renderer on Thouless ratios (repeated-measures ANOVA), *F*(1, 106) = 19.5, *p* ≪ 0.01 (Figure 9). Thouless ratios were consistently higher for Cycles images. Additionally, although we also found a significant effect of condition on mean Thouless ratio (repeated-measures ANOVA; *p* ≪ 0.01), the Cycles models seemed to rely on different cues than the EEVEE models, as shown by the significance levels in Figure 9 (paired *t*-test comparisons with the normal condition). In the normal, contrast, and shadows conditions, the Cycles models were essentially at perfect constancy, with average Thouless ratios of 0.97, 1.01, and 0.95, respectively, and no significant difference between them. The average Thouless ratios in the shading and shading & shadows conditions were significantly lower, at 0.87 and 0.78, respectively. This suggests that the Cycles models relied on object shading, and less so on shadows, to disentangle the reflectance and illuminance contribution to luminance, unlike the EEVEE models. Finally, the average Thouless ratio in the no-cue condition (0.07) was significantly greater than 0 (paired *t*-test, *p* ≪ 0.01). This last observation is particularly interesting, as in this condition there was no cue other than rendering noise that the model could use to estimate illuminance and reflectance. Taken together, these findings support the hypothesis that the Cycles models learned to exploit rendering noise to achieve lightness constancy.

## General discussion

Lightness perception is a long-standing area of research, and many studies have discovered important principles of lightness constancy (Adelson, 1993; Arend & Spehar, 1993; Gilchrist, 2006; Granzier & Gegenfurtner, 2012; Murray, 2021; Szafir et al., 2015; Wedge-Roberts et al., 2020). This research reveals lightness constancy to be a complex visual ability dependent on multiple factors, including local contrast, adaptation to the luminance range, and specular highlights, to name a few. Yet, despite these insights, few image-computable models of lightness perception exist.

Prior studies suggest that DNNs are promising candidates for such models, as they can match or even surpass human constancy levels on images of rich three-dimensional scenes (Flachot et al., 2022; Murray et al., 2023). Our work supports this view, as our model achieved supra-human levels of constancy. However, we identified critical differences in how humans and the model solve the task. In the scenes we used, human observers relied predominantly on local contrast to judge patch colors, whereas DNNs leveraged physically relevant cues like shadows, shading, and, when present, rendering noise that was correlated with illuminance.

One could argue from these findings that unconstrained DNNs are simply too powerful to be good models of human performance in this task. Our model learned to use physically relevant properties of the scenes we showed, and to worsen its constancy, we had to break the underlying physics by using composite scenes that silenced lighting cues from shadows and object shading. Human observers, not trained with these stimuli and possibly more limited in their capacity, relied on less computationally demanding cues like local contrast. On this view, in order to make the DNNs ‘more human’ we would have to limit their capacity, either by reducing their computational power (Kubilius et al., 2018) or by introducing additional constraints (Geirhos et al., 2018). Patel et al. (2025) also concluded that limiting networks’ abilities is an important part of making them good models of lightness perception. They found that a DNN like the present one was susceptible to several classic lightness illusions, even when images provided cues that might allow a sufficiently powerful observer to perceive reflectance correctly. Experiments with networks with a range of abilities (e.g., tendencies to use local or global information) may provide insight into what abilities support human lightness perception.

However, several other factors may explain the divergence between humans and the model. First, we deliberately limited the realism and complexity of the training set, to explore the role of generic features such as shading and occlusion. This resulted in a limited set of scene configurations (indoor), material types (Lambertian), object shapes (geometric), lighting configurations (two light sources), and shadow boundaries (sharp). Furthermore, the dynamic range was limited, and, in particular, specular highlights were excluded. The same can be said about motion and disparity. This simplified environment made many naturalistic cues unavailable, which may have been the reason we observed low Thouless ratios for human observers. These same factors may have made shadows and shading into more reliable cues that could be exploited by the network model, which was specifically trained on these scenes. More generally, the network was not subject to the same physical and biological constraints as human observers, nor was it asked to learn from the scene ensemble of a life time one avenue for constraining networks to rely less on shadows and shading would be to make these cues more difficult to exploit, for example, by increasing the complexity of the lighting, or by blurring or removing shadow boundaries. Under these conditions, the network might rely more heavily on local contrast, as we found for human observers. Another avenue would be to render a large dataset of visually richer scenes—with a higher dynamic range, more complex object shapes, and more varied illumination conditions—while preserving the same level of control that we have exploited here as we trained and tested our networks.

Second, full supervision during training, with access to pixelwise ground-truth values of reflectance and illuminance, is fundamentally different from how people learn to see the world (Anderson, 2015; Hoffman, Singh, & Prakash, 2015). Our visual system does not have direct access to the ground-truth about the properties of objects and surfaces. In the present work, we sought to understand whether a trained network has important similarities to human lightness perception, and this work has little to say about how this ability develops, in either humans or networks. However, if we are considering possible reasons for differences between humans and the network, this is certainly one area of interest. The loss function in our training routine was a mean squared error loss over the whole image, which may have encouraged the global strategy that the network seems to have developed. Furthermore, previous work has shown that fully supervised DNNs tend to develop strategies unlike those of human observers, more so than weakly or unsupervised training methods (Storrs et al., 2021). It is possible that a model trained in an unsupervised manner on the same images, using, for example, a contrastive loss (Chopra, Hadsell, & LeCun, 2005), would lead to more human-like strategies. This is a promising avenue for future work.

Finally, the human visual system must solve a much broader range of complex visual tasks than just learning correlates of reflectance and illuminance, such as object recognition, face recognition, and depth. Local contrast, although a valuable cue for human lightness constancy due to its invariance across changes in illuminance (Foster, 2011), is also crucial to some of these other visual functions. Indeed, early stages of the human visual system contain many contrast-sensitive cells, which are crucial for tasks like shape and object recognition (Derrington, Krauskopf, & Lennie, 1984; Wachtler, Sejnowski, & Albright, 2003). Contrast-sensitive cells are also thought to be necessary for the biological system to accommodate the high dynamic range of natural images within the smaller dynamic range of neural synapses (Brenner, Bialek, & Van Steveninck, 2000; Gaudry & Reinagel, 2007; Shapley & Victor, 1978; Shapley & Victor, 1981). Contrast-sensitive cells thus form the basis of human visual processing (Hoyer & Hyvärinen, 2000). From this point of view, our observers’ sensitivity to local contrast manipulations is unsurprising. This is very different from the training environment of our network model, which was optimized solely for reflectance–illuminance separation in artificial scenes with a low dynamic range where shadows and shading were highly diagnostic. Perhaps a better image-computable model of human lightness constancy would emerge from training simultaneously on a broader range of tasks, including shape and object recognition. Training such a model would require a large dataset of object-labeled naturalistic images taken under varying and known lighting conditions.

Another notable finding of the present work was the Cycles models’ reliance on ray-tracing noise that, within an image, is correlated with illuminance. In a different modeling context, Ding et al. (2019) reported the related result that if care is not taken, rendering noise can elevate the performance of trained classifiers. Given that ray-tracing is considered the most realistic rendering method (Shirley & Morley, 2008), it is the most commonly used method for generating large synthetic datasets (Li et al., 2018; Li et al., 2021; Roberts et al., 2021; Zhu et al., 2022). As a result, many models trained for inverse rendering and intrinsic image decomposition are trained on ray-traced datasets (Li & Snavely, 2018a; Li et al., 2020; Wang et al., 2021; Zhu et al., 2022) and may be susceptible to similar artifacts. This could limit their performance when they are applied to natural images, in which such artificial noise is not present. This issue is particularly relevant for models of human lightness and color constancy. To mitigate it, we suggest avoiding ray-tracing algorithms in favor of methods like rasterization, or potentially mixing ray-traced and rasterized images in training. Alternatively, recent years have seen the development of powerful de-noising methods (Bako et al., 2017; Chopra, Hadsell, & LeCun, 2005), and these methods might silence rendering noise as a cue for disentangling reflectance and illuminance. Regardless, in experiments that evaluate network performance, particularly when trained and evaluated on rendered scenes, we recommend including control conditions where good performance should be impossible, like our no-cue condition, as a test for the presence of unwanted cues.

Overall, our results show that a deep learning architecture trained for lightness constancy on complex rendered scenes is able to achieve a high level of constancy, but in a different manner than human observers. However, this does not discredit the potential of DNNs to model human lightness constancy or human vision in general. There are few image-computable models of human lightness perception, and the ones that have been developed fail often in complex scenes (Figure 4). Here we find the opposite problem: Our DNN can succeed even where human observers fail. DNNs are a powerful class of image-computable models, and our findings show that when images are carefully selected to exclude artifacts, DNNs can exploit relevant, naturalistic cues like shadows and shading. There are several factors that may explain the divergence we found between the network model and human observers, including network capacity, the realism of the training set, the kind of training, and the need to simultaneously learn several visual tasks. Exploring these factors is a promising approach for future work.

## Footnotes

1 Surface reflectance is the proportion of light that a surface reflects at each wavelength (McCluney, 2014). In the lightness literature, it is typically assumed to be constant over the visible spectrum and is thus specified by a single number. This is what we mean by *achromatic surface reflectance*, sometimes called “albedo” in the lightness literature. Achromatic surface reflectance is a physical property of spectrally non-selective surfaces. Lightness is perceived achromatic surface reflectance, and is a perceptual property (Gilchrist, 2006). It depends not only on the surface being judged, but also on the context in which the surface is seen.

2
https://github.com/AlbanFlachot/IID4constancy/tree/main.
